# Supplementary material for: MMSplice: modular modeling improves the predictions of genetic variant effects on splicing
Source: Genome Biol. 2019 Mar 1;20:48. doi: 10.1186/s13059-019-1653-z (PMC6396468; doi:10.1186/s13059-019-1653-z)
Supplement: Supplementary file 1 — Supplementary methods and figures. (PDF 674 kb) [file 13059_2019_1653_MOESM1_ESM.pdf]

MMSplice: modular modeling improves the predictions of genetic variant effects on splicing

1 Supplementary Methods

1.1 GTEx $\Delta\Psi_3$ and $\Delta\Psi_5$ dataset

For this benchmark, we restricted to variants that MaxEntScan can score, i.e. variants at less than 3 nt in the exon and less than 6 nt in the intron around donor sites in the case of Ψ_3, and variants at less than 3 nt in the exon and less than 20 nt in the intron around acceptor sites in the case of Ψ_5. Furthermore, we restricted the analysis to donor sites with only two supported alternative acceptor sites and to acceptor sites with only two alternative donor sites, according to the Human genome (hg19) alternative events v2.0 on the MISO website `https://miso.readthedocs.io/en/fastmiso/annotation.html`.

Next, we computed Ψ_3 and Ψ_5 with MISO for each alternative splicing event (with reference and alternative sequence), and averaged the Ψ_3 or Ψ_5 values from all samples with the same genotype. We required at least 2 supporting samples for homozygous variants and at least 6 supporting samples for heterozygous variants.

1.2 MMSplice competing splice site variants prediction

We show applying MMSplice to predict variant effect on $\Delta\Psi_5$ with arbitrary number of alternative splicing sites $n \in \{1...n\}$. We would like to predict variant effect on the Ψ_5 of exon i.

$$\Psi_{5_{\text{alt}}} = \sigma(\Delta\,\text{logit}(\Psi_5) + \text{logit}(\Psi_{5_{\text{ref}}})) \tag{1}$$

We can model $\Delta\,\text{logit}(\Psi_5)$ as follow:

$$\begin{aligned}\Delta\,\text{logit}(\Psi_5) &= \text{logit}\left(\frac{\exp\left(S_i^{\text{alt}}\right)}{\sum_{j=1}^{n}\exp\left(S_j^{\text{alt}}\right)}\right) - \text{logit}\left(\frac{\exp\left(S_i^{\text{ref}}\right)}{\sum_{j=1}^{n}\exp\left(S_j^{\text{ref}}\right)}\right) \\ &= S_i^{\text{alt}} - S_i^{\text{ref}} - \left(\log\left(\sum_{j=1,j\neq i}^{n}\exp\left(S_j^{\text{alt}}\right)\right) - \log\left(\sum_{j=1,j\neq i}^{n}\exp\left(S_j^{\text{ref}}\right)\right)\right)\end{aligned} \tag{2}$$

Where S_j^{ref} is the MMSplice score for the *j-th* alternative exon with reference sequence, and S_j^{alt} is the MMSplice score for the *j-th* alternative exon with alternative sequence. $\text{logit}(\Psi_{5_{\text{ref}}})$ is the logit of the Ψ_5 measured by MISO from the reference sequence.
In the case of two alternative splice sites as considered in the GTEx data, the above equation 2 equals to:

$$\begin{aligned}\Delta\,\text{logit}(\Psi_5) &= (S_1^{\text{alt}} - S_1^{\text{ref}}) - (S_2^{\text{alt}} - S_2^{\text{ref}}) \\ &= \Delta S_1 - \Delta S_2\end{aligned} \tag{3}$$

Which is the formula given in the main text.
We then calculate the predicted $\Delta\Psi_5$ as for homozygous and heterozygous variants as follow:

$$\begin{aligned}\Delta\Psi_{5_{\text{homo}}} &= \Psi_{5_{\text{alt}}} - \Psi_{5_{\text{ref}}} \\ \Delta\Psi_{5_{\text{hetero}}} &= (\Psi_{5_{\text{ref}}} + \Psi_{5_{\text{alt}}})/2 - \Psi_{5_{\text{ref}}}\end{aligned} \tag{4}$$

COSSMO model was applied to the reference sequence and alternative sequence separately. $\Delta\Psi_5$ for homozygous and heterozygous variants are calculated as):

$$\begin{aligned}\Delta\Psi_{5_{\text{homo}}} &= \Psi_{5_{\text{alt}}} - \Psi_{5_{\text{ref}}} \\ \Delta\Psi_{5_{\text{hetero}}} &= (\Psi_{5_{\text{alt}}} + \Psi_{5_{\text{ref}}})/2 - \Psi_{5_{\text{ref}}}\end{aligned} \tag{5}$$

Analogous equations apply to differences in Ψ_3.
MaxEntScan model was applied to the reference sequence and alternative sequence separately. MaxEntScan predicted variant effect is:

$$\begin{aligned}\Delta\Psi_{5_{\text{homo}}} &= (S_{2_{\text{alt}}} - S_{1_{\text{alt}}}) - (S_{2_{\text{ref}}} - S_{1_{\text{ref}}}) \\ \Delta\Psi_{5_{\text{hetero}}} &= ((S_{2_{\text{alt}}} - S_{1_{\text{alt}}}) + (S_{2_{\text{ref}}} - S_{1_{\text{ref}}}))/2 - (S_{2_{\text{ref}}} - S_{1_{\text{ref}}})\end{aligned} \tag{6}$$

1.3 ClinVar variant pathogenicity prediction

To predict variant pathogenicity, every model, including ensemble ones were applied by training a logistic regression model on their predicted scores. Detailed list of features used for other models are provided by [1]. For the MMSplice model, the following features were included:

- Intron 3′module delta score
- Acceptor module delta score
- Exon 5′module delta score
- Donor module delta score
- Intron 5′module delta score
- Indicator variable: Intron 3′module overlap with acceptor module
- Indicator variable: Exon 5′module overlap with acceptor module or donor module
- Indicator variable: Intron 5′module overlap with donor module

For MutPred Splice model, following features were included:

- MutPred Splice score
- Indicator variable: MutPred Splice score is NA

The same pre-processing pipeline was applied to all models. If a variant was not scored by a model, then its differential effect for this model was predicted to be 0. Moreover, all features were standardized to have mean zero and variance one.

References

[1] Ziga Avsec, Roman Kreuzhuber, Johnny Israeli, Nancy Xu, Jun Cheng, Avanti Shrikumar, Abhimanyu Banerjee, Daniel S Kim, Lara Urban, Anshul Kundaje, Oliver Stegle, and Julien Gagneur. Kipoi: accelerating the community exchange and reuse of predictive models for genomics. *bioRxiv*, 2018.

2 Supplementary figures

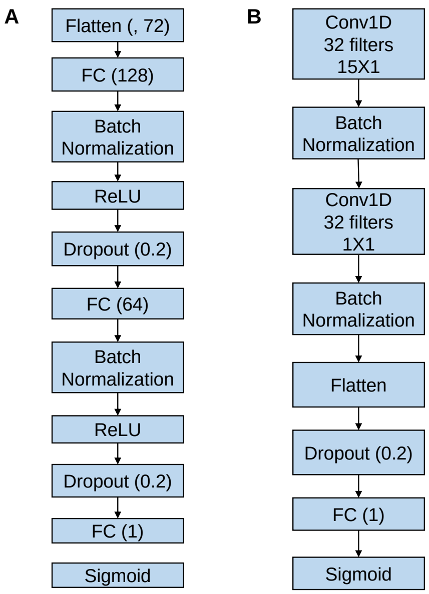

Fig. S1: Model architecture of donor (**A**) model and acceptor (**B**) model.

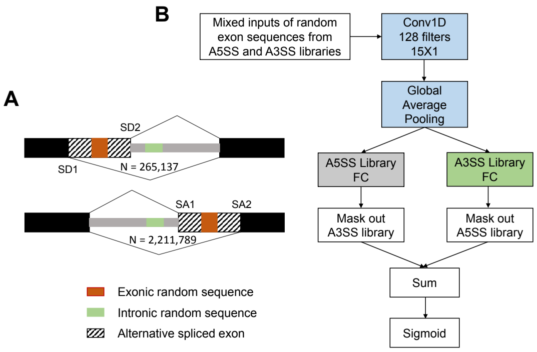

Fig. S2: Exon scoring models. Intron scoring models were trained with the same principle with the input being intronic random sequences. (**A**) Two libraries of the splicing MPRA experiment. A5SS (up) and A3SS (down). (**B**) Model architecture for exon scoring module. The first convolution layer was shared across two libraries. The dense layers were learned specifically for each library.

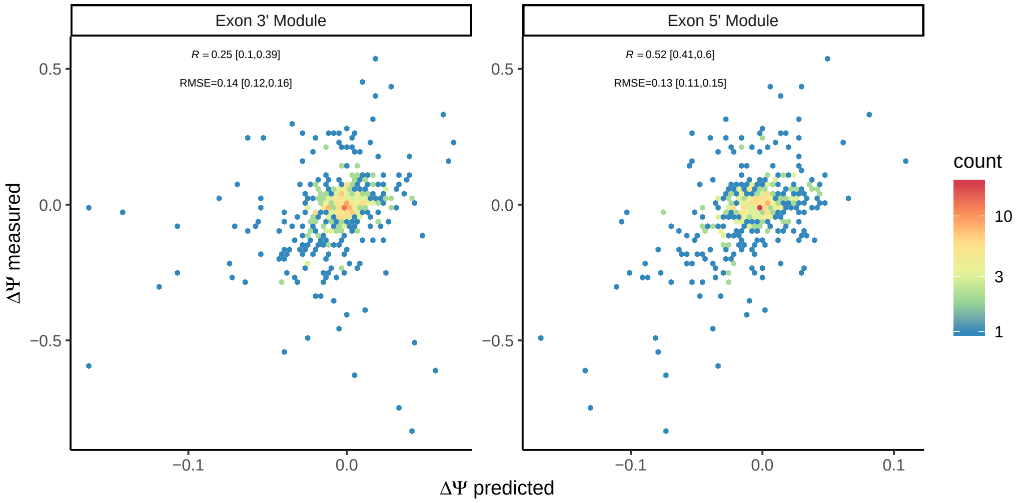

Fig. S3: Measured differences in Ψ (y-axis) versus predicted differences (x-axis) for the MMSplice exon 3'module (left) and for the MMSplice exon 5'module (right) for the training exonic variants of the Vex-seq dataset.

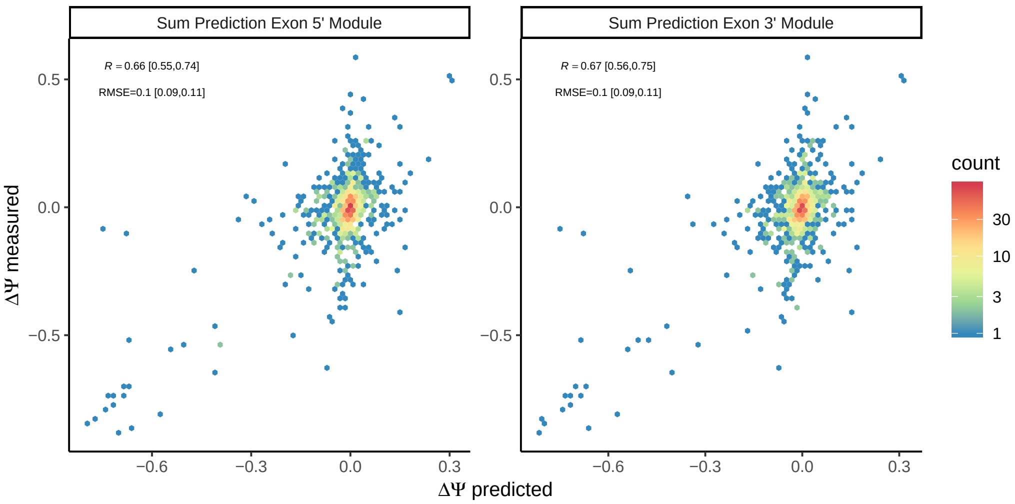

Fig. S4: Measured differences in Ψ (y-axis) versus predicted differences (x-axis) when summing the predicted scores of the MMsplice donor site modules, intronic modules and the exon 5' module (left), and when summing the predicted scores of the MMsplice donor site modules, intronic modules and the exon 3' module (right). The plots show the training variants of the Vex-seq dataset.

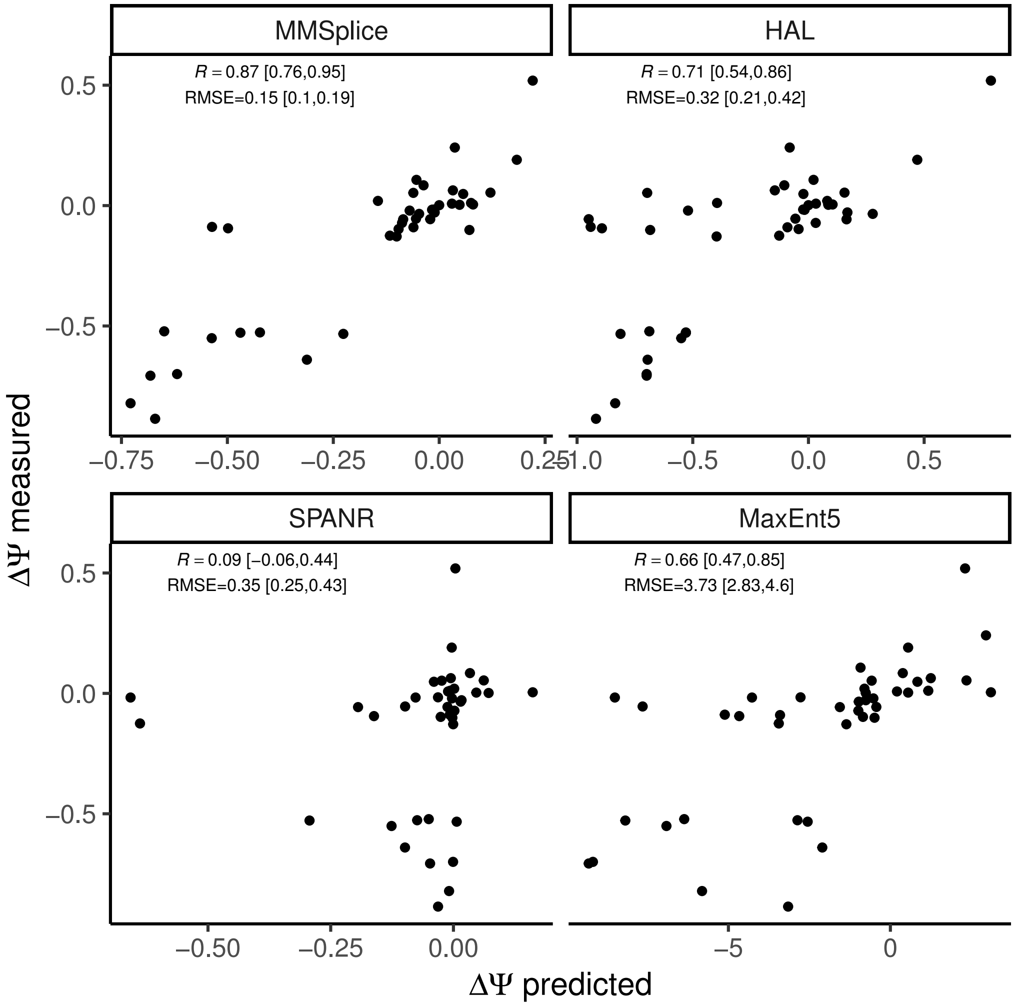

Fig. S5: Measured differences in Ψ (y-axis) versus predicted differences (x-axis) for MMSplice, HAL, SPANR and MaxEntScan5 for variants of the Vex-seq test data within 3 nt in the exon and 6 nt in the intron around the donor sites.

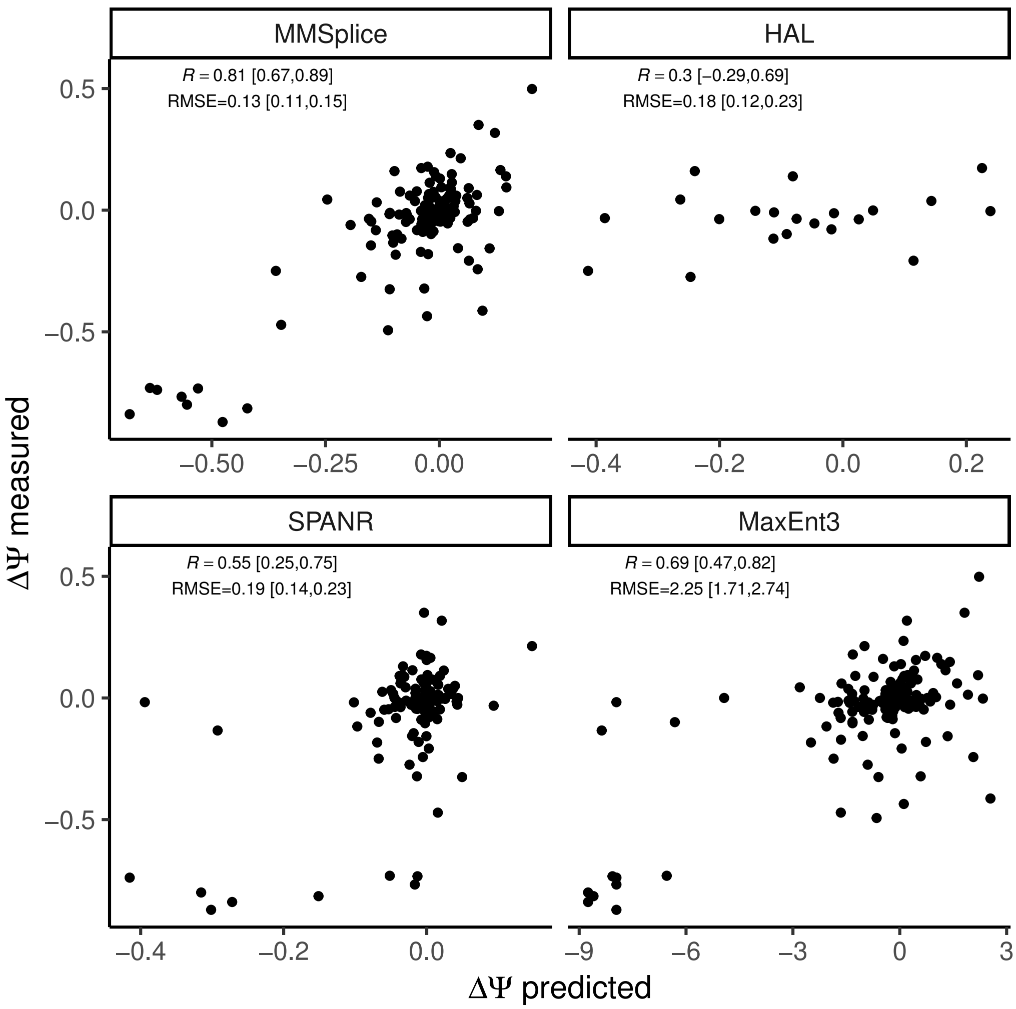

Fig. S6: Measured differences in Ψ (y-axis) versus predicted differences (x-axis) for MMSplice, HAL, SPANR and MaxEntScan5 for variants of the Vex-seq test data within 3 nt in the exon and 20 nt in the intron around the acceptor sites. HAL only scores the 3 nt in the exon, but no intronic variants.

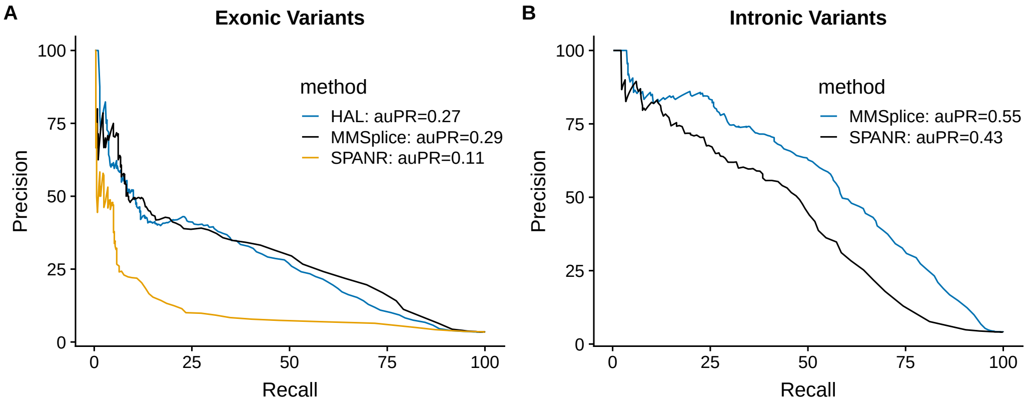

Fig. S7: Model benchmarking on MFASS data. (**A**) Precision-recall curve for MMSplice (black), HAL (blue) and SPANR (orange) for MFASS exonic variant. (**B**) Precision-recall curve for MMSplice (blue), SPANR (black) for intronic variants.

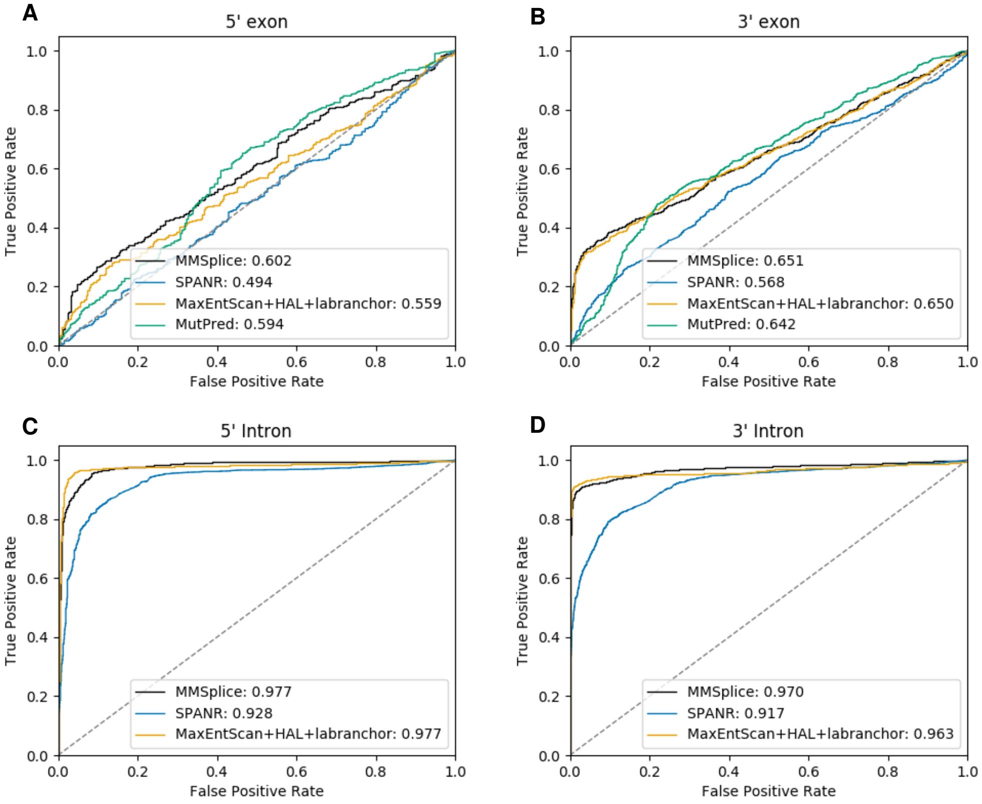

Fig. S8: Comparing (1) MMSplice, (2) SPANR, (3) MutPred SPlice, (4) the ensemble model with MaxEntScan, HAL and LaBranchoR on classifying "pathogenic" versus "benign" variants from ClinVar. ROC curve computed with ClinVar variants from 4 different regions.

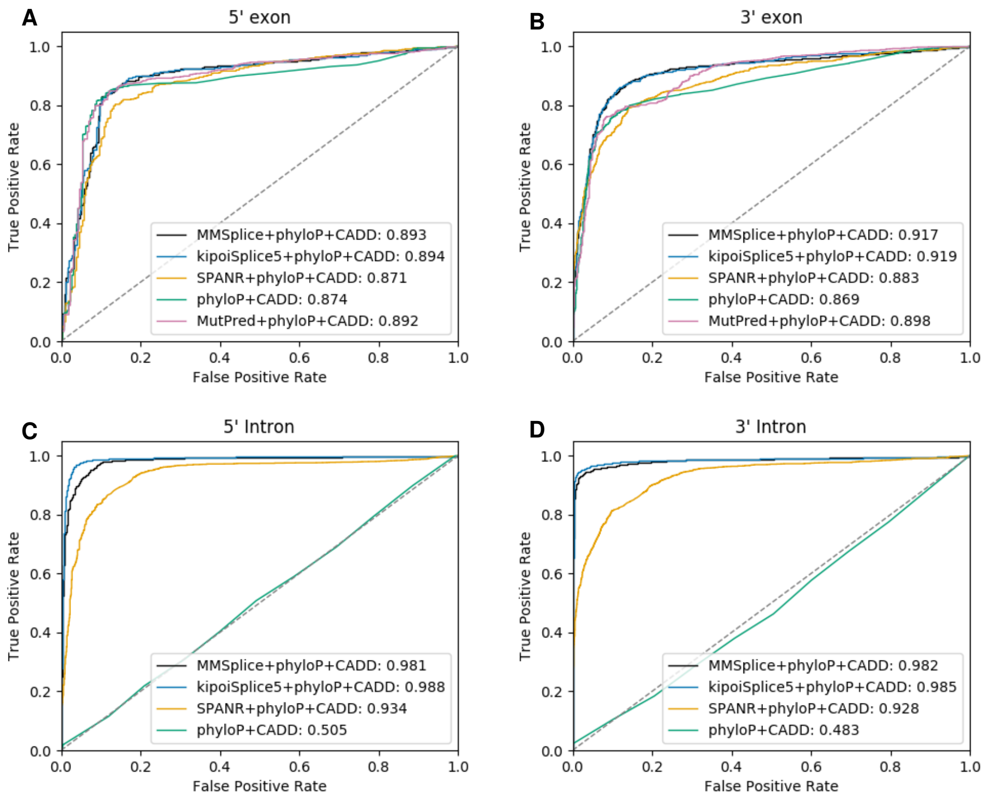

Fig. S9: Comparing three ensemble models that used phyloP and CADD as features on classifying "pathogenic" versus "benign" variants from ClinVar: (1) ensemble of MMSplice with phyloP and CADD (MMSplice+phyloP+CADD), (2) ensemble of MaxEntScan, HAL, LaBranchoR, MMSplice with phyloP and CADD (kipoiSplice5+phyloP+CADD), (3) ensemble of SPANR with phyloP and CADD (SPANR+phyloP+CADD), (4) ensemble of phyloP and CADD (phyloP+CADD), (5) ensemble of MutPred with phyloP and CADD (MutPred+phyloP+CADD). ROC curve computed with ClinVar variants from 4 different regions.

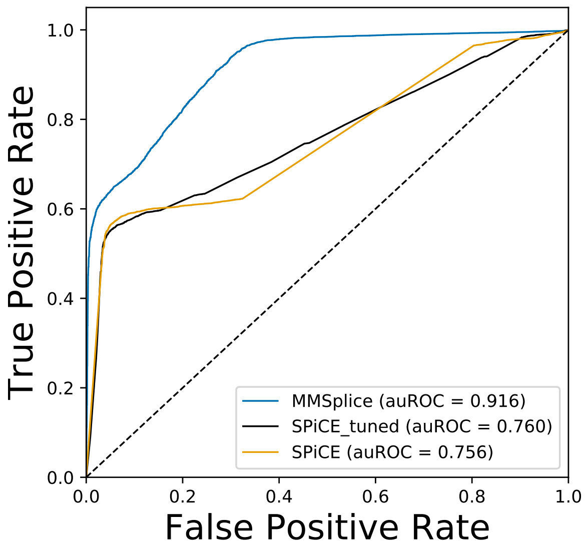

Fig. S10: ROC curve comparing MMSplice (blue) to SPiCE (orange) and a SPiCE model that we fitted to the ClinVar dataset (SPiCE_tuned, black). The calculation of the ROC curve was restricted to the 13,820 ClinVar variants that SPiCE was able to score.
